# Prediction of hyaluronic acid target on sucrase-isomaltase (SI) with reverse docking and molecular dynamics simulations for inhibitors binding to SI

**DOI:** 10.1371/journal.pone.0255351

**Published:** 2021-07-30

**Authors:** Xiao Li, Keqing Qian, Weiwei Han

**Affiliations:** 1 Engineening Research Center of Chinese Ministry of Education for Edible and Medicinal Fungi, Jilin Agricultural University, Changchun, Jilin, China; 2 Key Laboratory for Molecular Enzymology and Engineering of Ministry of Education, School of Life Science, Jilin University, Changchun, China; University of Calgary, CANADA

## Abstract

*Auricularia cornea (E*.*)* polysaccharide is an important component of *A*. *cornea Ehrenb*, a white mutant strain of *Auricularia* with biological activities, such as enhancement of human immune function and cancer prevention. The hyaluronic acids (HAs) are important components of the *A*. *cornea* polysaccharide and have extremely high medicinal value. In this study, we used HA to search the target protein sucrase-isomaltase (SI). In addition, we also performed molecular dynamics (MD) simulations to explore the binding of three inhibitors (HA, acarbose and kotalanol) to SI. The MD simulations indicated that the binding of the three inhibitors may induce the partial disappearance of α helix in residues 530–580. Hence, the hydrogen bond for Gly570-Asn572, which was near the catalytic base Asp471 in SI, was broken during the binding of the three inhibitors. We reveal a new inhibitor for SI and provide reasonable theoretical clues for inhibitor binding to SI.

## Introduction

*Auricularia auricula* is an edible and medicinal fungus ranking fourth in production among the worldwide [1–4]. Scientific research is extensive on this species because of the abundant resources of A. auricula in China [5, 6]. In 2005, Lin *et al*. found a new a kind of white variant *Auricularia* fungus named as *A*. *fucosuccinea*. In 2017, Dai *et al*. named this white variant as *A*. *cornea Ehrenb* [7]; the variant is highly nutritious and has high economic value [8].

In 2018, Li Yu *et al*. pointed out that *A*. *cornea Ehrenb* suppressed the levels of total cholesterol and triglyceride and enhanced levels of hepatic glycogen and high-density lipoprotein cholesterol, which may be involved in diabetes mellitus (DM) [7], which is a progressive metabolic disease [9].

The polysaccharide of *A*. *cornea Ehrenb* has been widely studied; it is an important chemical component for regulating human life activities. The polysaccharide content of *A*. *cornea Ehrenb* is reportedly better than other *Auricularia* species [7]. *A*. *cornea Ehrenb* polysaccharide is widely used in medicine, food science and other fields. However, we still do not know which components in the polysaccharide of *A*. *cornea Ehrenb* are involved in metabolic disease.

Hyaluronic acid (HA) is the important compound of polysaccharide of *A*. *cornea Ehrenb*. HA belongs to the glycosamino glycans connected with β 1,4 glycosidic bond. Considering its unique physical and chemical properties, HA is widely used in medicine [10–12]. So, in this study, HA was used to search the target protein in metabolic disease. Sucrase-isomaltase (SI) functioned as an attractive target for inhibition by α-glucosidase inhibitors as a means of controlling blood glucose levels in individuals with type 2 diabetes [13, 14]. In addition, we also used molecular dynamics (MD) simulations to explore the binding mode of the three inhibitors (HA, acarbose and kotalanol) to SI. Our results will provide useful clues for further *A*. *cornea Ehrenb* study.

## Methods

### Reverse docking

The 2D structure of HA was used for SwissTargetPrediction [15] to search for the target protein.

### Protein preparation

The 3D structure of the SI was obtained from the RCSB Protein Data Bank (www.rcsb.org) (PDB ID: 3LPO) [13]. The 3D structures of HA, acarbose and kotalanol were downloaded from the Chemspider database (www.chemspider.com). In this study, AutoDock Vina [16] was used to construct protein-inhibitor complexes. In the AutoDock Vina configuration files, the parameter num_modes was set to 9 Å. We identified the receptor binding pocket based on the point of the substrate binding to the SI. Hence, we kept all the rotatable bonds in ligands flexible during the docking procedure, and we kept all the protein residues inside the binding pockets rigid. The Kollman charges were used to convert all receptors and ligands to the PDBQT format using the AutoDockTools package [17]. The search spaces were 30 × 30 × 30 Å. The docking results were clustered automatically.

### Molecular dynamics simulations

All MD simulation courses were conducted by using the Amber 16 package [18]. The AMBER ff99SB forcefield [19] was applied to the SI-inhibitor complexes. Then, they were solvated using the TIP3P water model [20] with the box at 12 Å. The Counter ions (Na+ and Cl-) were assigned to neutralise the three systems. Based on each of the prepared systems, energy minimization was used for the solvent complexes. Then, the constraints were used on protein backbone atoms. During the energy minimisation, the steepest descent algorithm [20] and conjugate gradient algorithm [21] were used successively. Subsequently, each system underwent a gradual heating process for 500 ps from 0 to 300 K and then was equilibrated for at 300 K for another 500 ps. Finally, the whole system was performed 100 ns MD simulation at 1 atm constant pressure and 300K constant temperature conditions. We used the Langevin Nosé–Hoover thermostat [22, 23] and the Parrinello–Rahman method [24, 25] to maintain a constant temperature and a constant pressure of 1 atm in each system. The LINCS algorithm were constrained by all bonds [26]. The long-range electrostatic interactions were performed with the particle mesh Ewald method [27] with a grid size of 1.2 Å. The periodic boundary conditions were implemented in all directions along the simulation box.

### Data analyses

PCA was performed using Bio3D version 2.3.0 to study the collective motions in 100 ns of protein-ligand complex [28, 29]. This method used the calculation and diagonalisation of the covariance matrix. The covariance matrix was calculated as follows:

Cij=<(xi‐<xi>)(xj‐<xj>)> (1)

where xi/xj is the coordinate of the ith/jth atom of the systems. Free energy landscape (FEL) is a map of all possible conformations of molecular entities [30] and can be used to understand the stability, folding and function of the protein. The FEL can be constructed as follows:

ΔG(X)=‐KBTlnP(X) (2)

where KB is Boltzmann constant, T is the temperature of simulation systems, and 300 K is set in the current calculations. P(X) is the probability distribution of the molecular system along the PCs.

### MM-PBSA calculations

Molecular mechanics Poisson-Boltzmann surface area (MM/PBSA) is a popular method to calculate the binding free energy between protein and ligands. It is more accurate than most scoring functions of molecular docking and less computationally demanding than alchemical free energy methods with Amber 16 package [18].

The Molecular Mechanics-Generalised Born Surface Area (MM/GBSA) method in AMBER16 package [18] was used to calculate the binding free energies. A total of 200 snapshots were chosen evenly from the MD trajectory.

## Results and discussion

### Reverse docking

Firstly, HA was used to search for target protein with SwissTargetPrediction [15]. Among these results, the possibility of binding score between the target proteins and HA is not very different. SI is an attractive target for inhibition by α-glucosidase inhibitors to control blood glucose levels related to type 2 diabetes [14]. In 2018, Li Yu *et al*. pointed out that *A*. *cornea Ehrenb* was involved in DM [7], a progressive metabolic disease [9]. In addition, HA is a disaccharide; so, SI was selected for further study.

### Binding pose of the inhibitors to SI

**Fig 1:**
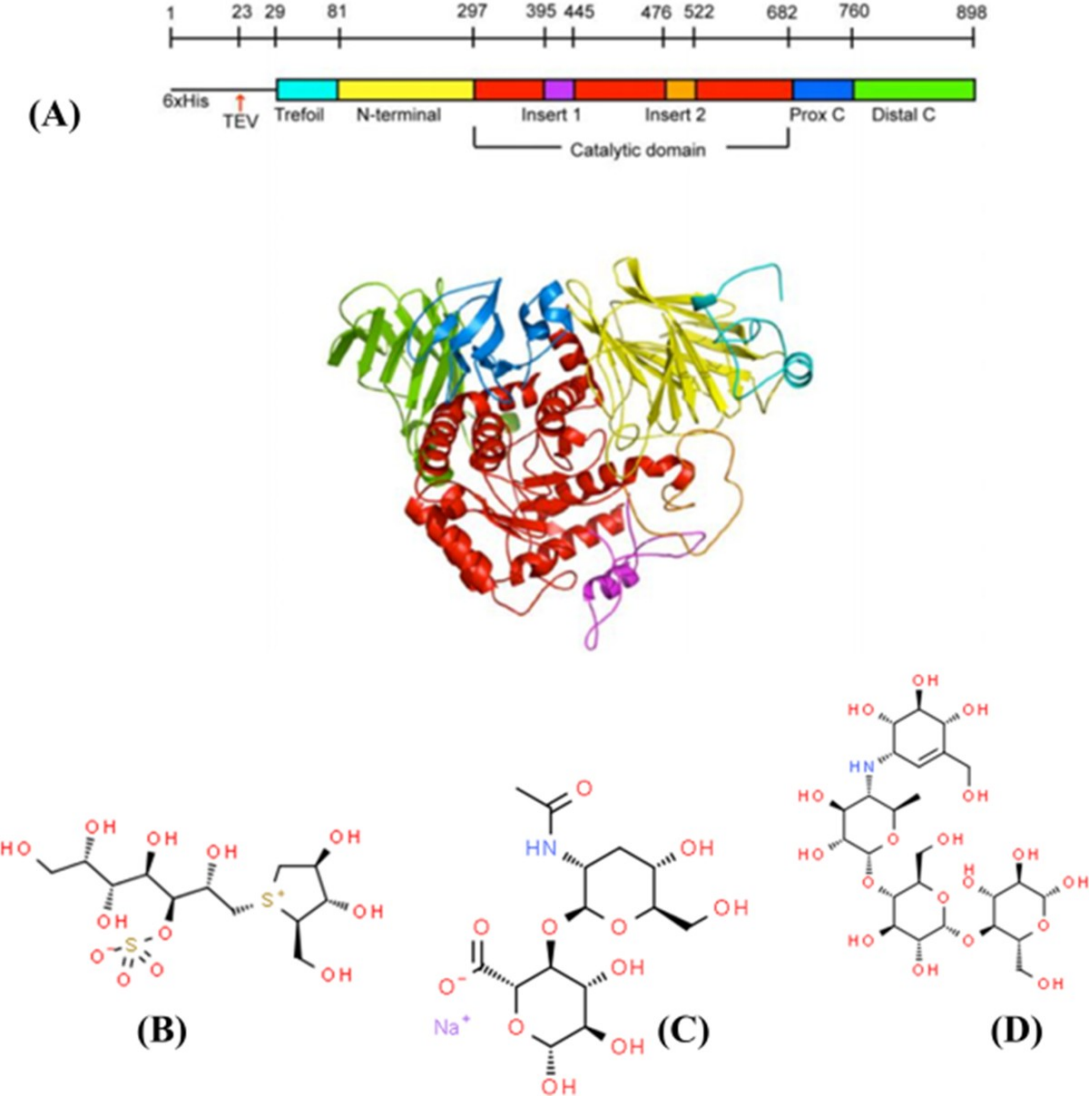
(A) The 3D structure of SI (PDB code 3LPO). (B) the disaccharide base of kotalanol. (C) The disaccharide base of acarbose. (D) The disaccharide base of HA.

SI is composed of the N- and C-terminal duplicated catalytic domains (Fig 1A). The N-terminal catalytic domain of N-terminal SI has a broader specificity for both 1,4- and 1,6-oligosaccharides [13]. Acarbose and kotalanol (the known α-glucosidase inhibitors) [13] and HA in Fig 1B–1D are docked with AutoDock Vina. The complex with SI bound with kotalanol were upload from PDB (PDB code 3LPP). The SI- kotalanol interaction was shown in S1A Fig. We also docked kotalanol to SI (S1B Fig). It can been seen that the docked kotalanol was similar to the crystal kotalanol, which indicated the docking software, AutoDock Vina, was reliable.

**Fig 2:**
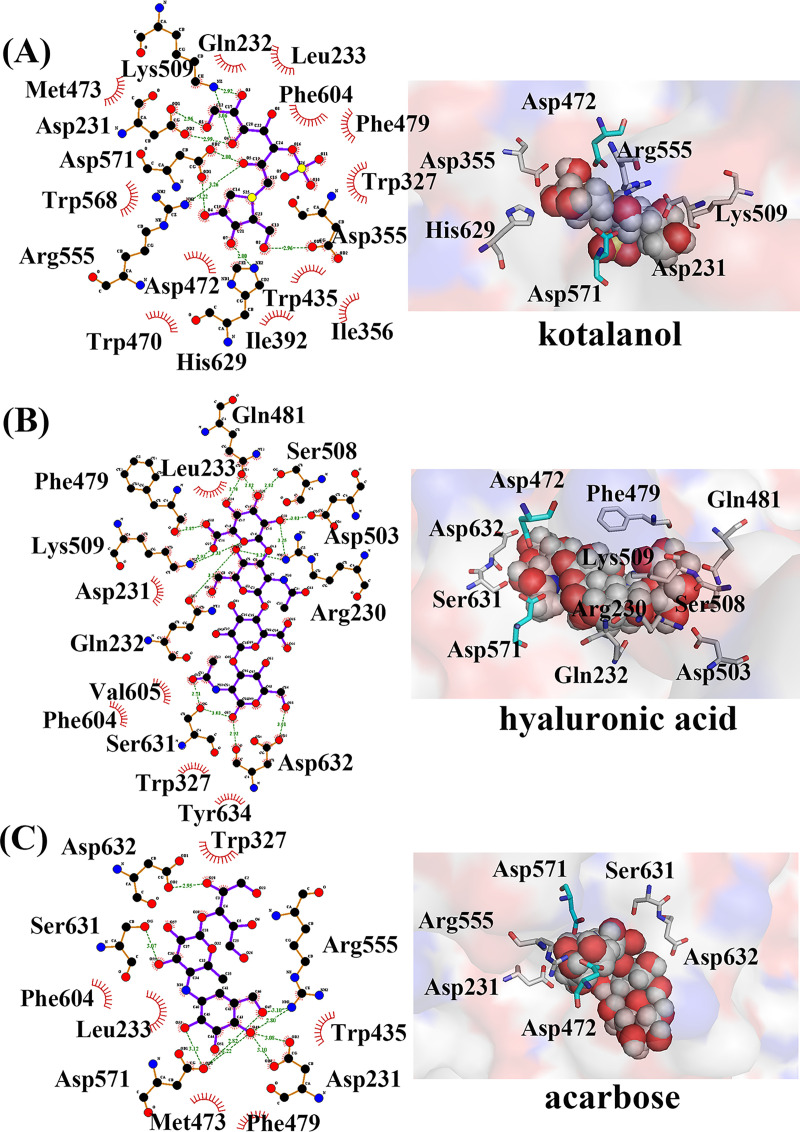
(A) The docking pose of SI-acarbose. (B) The docking pose of SI-kotalanol. (C) The docking pose of SI-HA.

According to Fig 2A–2C, it can be concluded that the disaccharide base of acarbose, kotalanol and HA was located at an active site. According to the SI-acarbose complex in Fig 2A, the residues Asp632, Trp327, Arg555, Trp435, Asp231, Phe479, Met473, Leu233, Asp571, Phe604 and Ser631 were related to acarbose binding. In particular, Asp632, Srer631, Asp571, Arg555 and Asp231 made a hydrogen bond with acarbose, and hence, they are possibly important residues for acarbose binding to SI. Fig 2B shows that residues Glu232, Leu233, Phe604, Phe479, Trp327, Asp355, Trp435, Ile356, Ile392, His629, Asp472, Trp470, Arg555, Asp571, Asp231 and Met473 were important residues for kotalanol binding. His629, Asp355, Asp571, Arg555, Asp231 and Lys509 formed hydrogen bonds with kotalanol. Fig 2C shows that Gln481,Ser508, Asp503, Arg230, Asp632, Tyr634, Trp327, Ser631, Val605, Phe604, Gln232, Asp231, Lys509, Phe429 and Leu233 were around HA binding. In particular, Gln481, Ser508, Asp503, Arg230, Lys509,Glu232, Phe429, Asp632 and Ser631 were anchor residues, because they made a hydrogen bond with HA. The three inhibitors were all in the catalytic (α/β)_8_ barrel subdomain (residues 297–681). Seen from S1B–S1D Fig, the docked kotalanol and acarbose had similar pose with crystal kotalanol. They are all in the same active pocket. So, the three complexes were reliable and can be used for further study.

### Conformational changes for the inhibitors binding to SI

**Fig 3:**
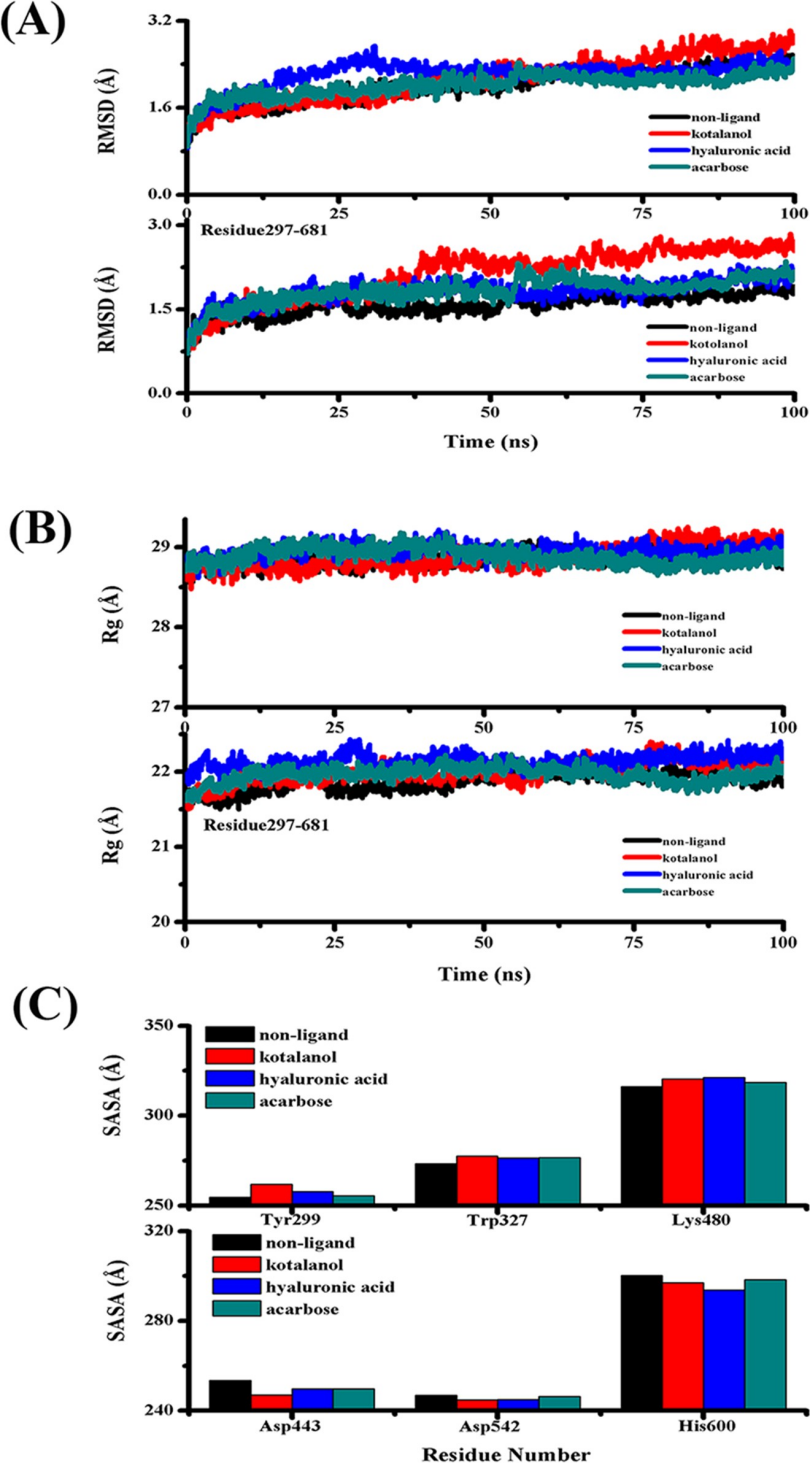
(A) The RMSD plot during MD simulations. (B) The Rg plot during MD simulations. (C) The SASA score.

To explore the conformational changes for the inhibitors binding to SI, MD simulations for the four systems (SI, SI-acarbose, SI-kotalanol and SI-HA) were performed with Amber 16 software [18]. The parameters of MD simulations are listed in Table 1. According to Fig 3A (RMSD plot), all four complexes were stable. MD simulation for free SI at a trajectory of 100 ns and with RMSD value of 2.4 Å was used as a reference. According to the curves where SI combined with any inhibitor, the value of RMSD became lower than that in free protein in the catalytic domain (residues 297–681). SI with kotalanol had a more drastic score that other inhibitor-enzyme complexes either in the full protein or in the catalytic domain (residues 297–681). Our results indicated that the conformational changes in a protein were more than those that occurred in a protein with inhibitors. When SI combined with an inhibitor, the conformation became stable. Kotalanol is a better inhibitor with lower Ki compared with the others, and so, it may induce larger conformational changes in SI [13].

**Table 1:** The number of atoms in different systems.

	non-ligand	kotalanol	acarbose	HA
**protein**	13740	13740	13740	13740
**ligand**	0	50	87	97
**ions(Na**^**+**^**)**	25	25	25	25
**water**	93408	93399	93351	93318
**total**	107173	107214	107203	107180

The rigidity of the protein system was examined using R_g_ values. The R_g_ plot of the α-carbon atoms versus time was obtained and presented in Fig 3B. The R_g_ values retained their stability throughout the 100 ns time of the MD simulation, which corresponded to the simulation. These values were used to facilitate the interpretation of the secondary structure. The R_g_ value for four systems (in full protein) was stable at about 28 Å, whereas the four systems, i.e., the catalytic domain (residues 297–681), were stable at about 21 Å. The three inhibitors made the secondary structure of the catalytic domain (residues 297–681) more stable and compact.

The overall conformational changes were further validated by the SASA graph, which was plotted against the MD simulation time, as shown in Fig 3C. The probabilities based on the SASA plots indicated that the four complexes had similar values. However, in Fig 3D, the SASA score of Tyr299, Trp327 and Lys480 were shown to be lower than that in the free SI. At the same time, Asp443, Asp542 and His600 had higher SASA score than SI. All six residues were located at the active site. These changes may cause some movement for inhibitor binding.

**Fig 4:**
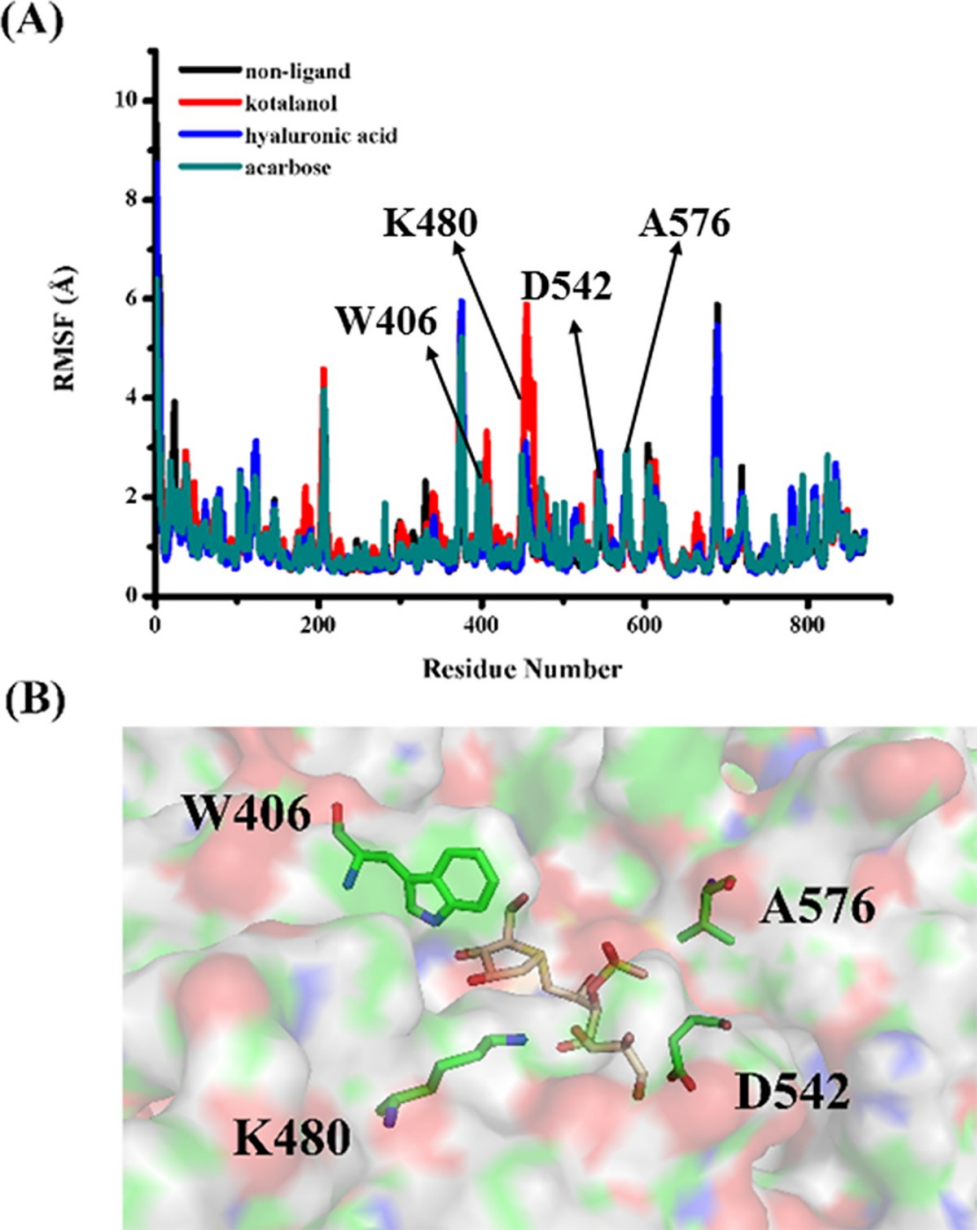
(A) RMSF plot during MD simulations. (B) the active residues for inhibitors binding.

Fig 4A shows the RMSF plot, which was calculated to evaluate the protein flexibilities when it combined with the inhibitor or without inhibitors. The observed fluctuations were local and limited to the modification sites. The Fig 4A indicated that free protein amino acid residues at the positions of 406, 480 and 542–576 fluctuated relative to the others and in comparison with the other curves. The amino acid residue fluctuations for combined inhibitor protein were stable and similar during the MD simulation. Fig 4B shows the active residues Trp406, Lys480, Ala576 and Asp542.

**Fig 5:**
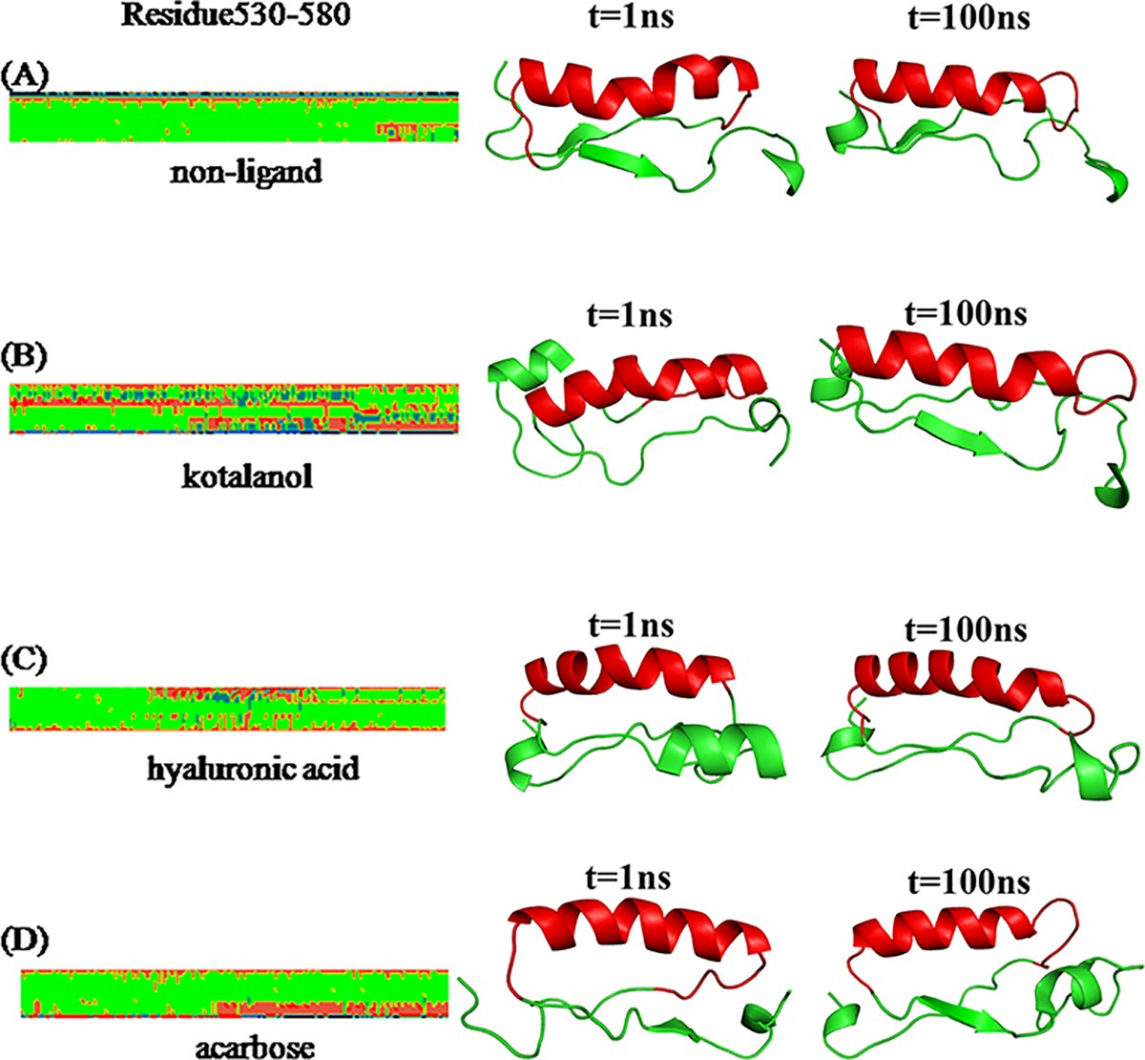
Differences in the secondary structures of residues Gly530 to Glu580 of (A) SI. (B) SI-kotalanol. (C) SI-HA. (D) SI-acarbose.

To explore the secondary structural changes related to the binding of three inhibitors, secondary structures during MD simulations were calculated, as shown in Fig 5A–5D. Fig 5A–5D shows the location of residue Gly530 to Glu580. The conformational changes caused by the three inhibitors were investigated and compared with those caused by the free SI. To investigate the conformational changes, we obtained the difference in the secondary structure (DSSP). The DSSP of residues Gly530 to Glu580 differed from that in the SI-inhibitor. The α helix of residues Gly530 to Glu580 in SI-inhibitors partly disappeared.

Among them, we truncated the most different parts, as presented in Table 2. In residues Gly530 to Glu580, free SI had a probability of 86.50%–100.00%, whereas the probabilities of the other systems were almost decreased. As Gly530 to Glu580 belongs to the catalytic domain (residues 297–681), so the conformation change on the 530–580 domain induced by inhibitors binding may be the main factor affecting the efficiency of enzyme catalysis.

**Table 2:** The probability (%) for alpha (residue 530–580) of the four systems during MD simulations.

	W547	E548	Q549	M550	E551	G556	M557	L558	E559	F560
non-ligand	86.50	87.00	93.00	95.00	89.50	100.0	100.0	100.0	99.50	99.50
kotalanol	39.00	39.00	44.00	44.00	43.00	14.00	24.50	26.50	47.50	65.50
hyaluronic acid	27.00	30.50	62.50	63.00	62.00	98.50	99.50	86.00	86.00	94.50
acarbose	40.00	40.50	43.00	50.00	60.00	98.00	99.50	99.50	98.00	98.00

**Fig 6:**
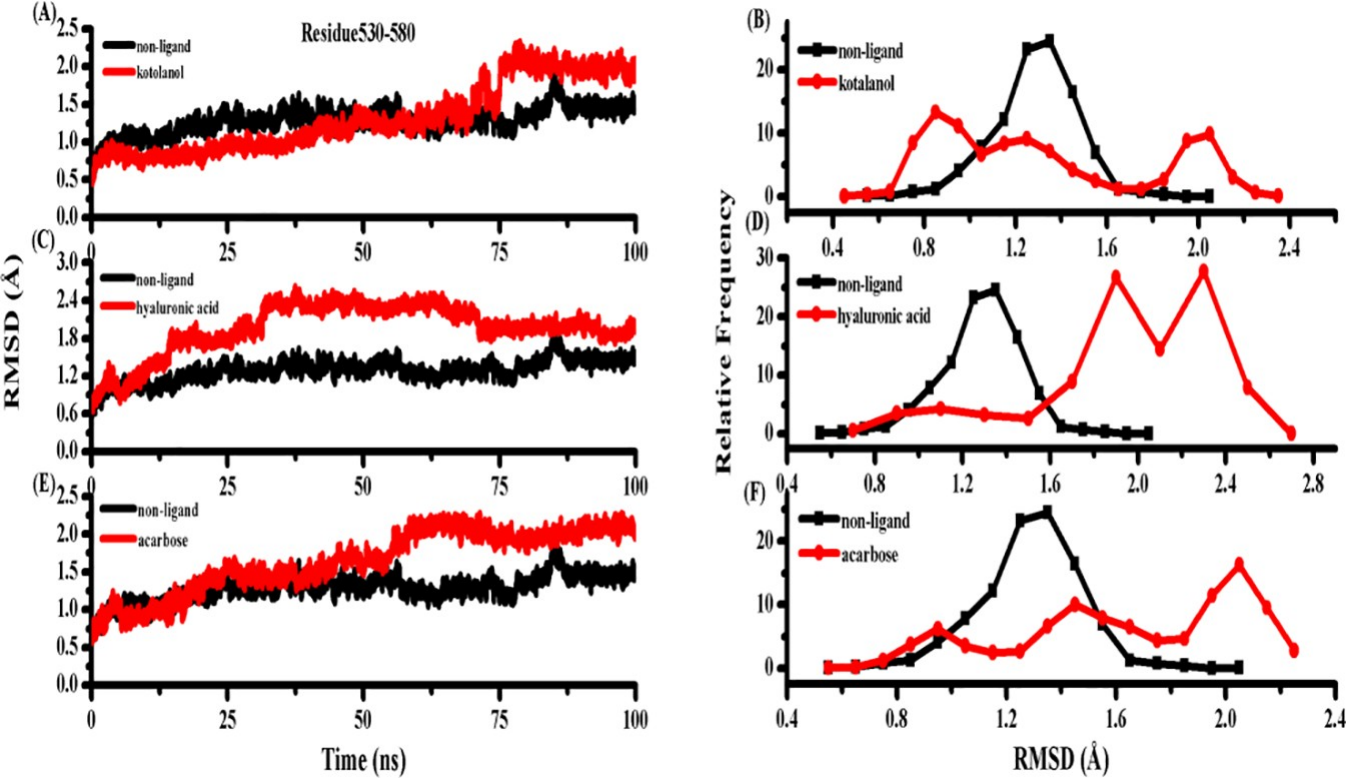
(A) RMSD plot of residue 530–580 between free SI and SI-kotalanol. (B) the relative frequency of RMSD plot of residue 530–580 between free SI and SI- kotalanol. (C) RMSD plot of residue 530–580 of free SI and SI-HA. (D) the relative frequency of RMSD plot of residue 530–580 between free SI and SI- HA. (E) RMSD plot of residue 530–580 between free SI and SI-acarbose. (F) the relative frequency of RMSD plot of residue 530–580 between free SI and SI-acarbose.

According to Fig 6A, 6C and 6E, free SI stayed stable within the 1.5 Å RMSD value. However, the RMSD value of the three inhibitor-enzymes were kept at a high level, showing that the three inhibitors binding to SI induced Gly530 to Glu580 domain flexibly. Fig 6B, 6D and 6F show the relative frequency of RMSD plot of Gly530 to Glu580 domain for the free SI with the SI-inhibitor complex. Thus, the three inhibitors binding to SI caused drastic changes in the Gly530 to Glu580 domain. Residues Gly530 to Glu580 contained the acid/base catalyst, Asp571.

**Fig 7:**
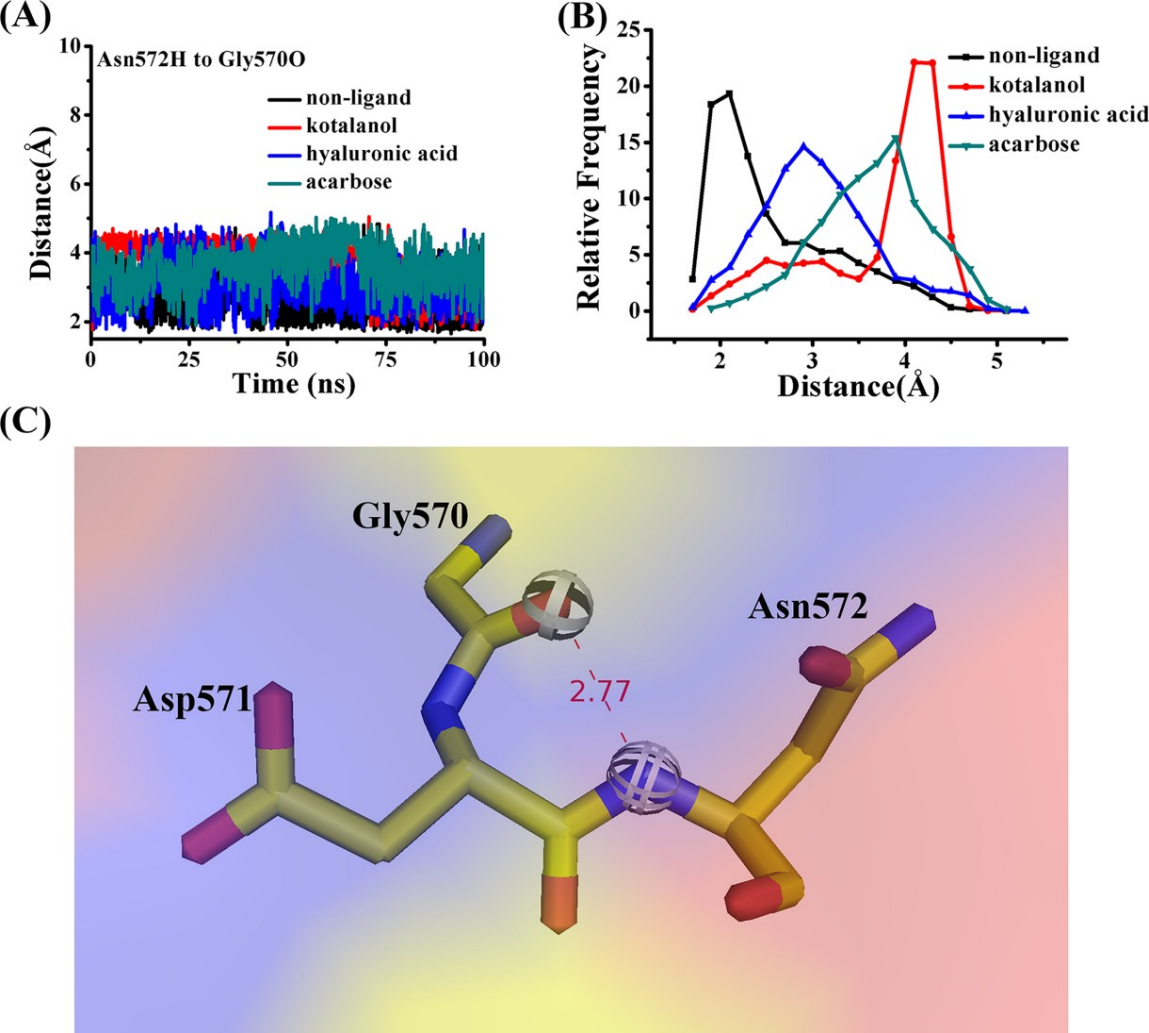
(A) The distance between Asn572H and the O atom of Gly570. (B) The relative frequency of distance between Asn572H and the O atom of Gly570 of the four complexes. (C) The hydrogen bond between Asn572H and the O atom of Gly570.

Seen from Fig 7A, Asn572 made hydrogen bonds with Gly570. The relative frequency of distance between Asn572 and the O atom of Gly570 of the four complexes are shown in Fig 7B. The hydrogen bond between Asn572 and Gly570 contained all the MD simulations (about 2 Å), whereas this may become weak in the inhibitor-SI complexes (SI-HA, about 3 Å), or it disappeared. Residues Gly530 to Glu580 contained acid/base catalyst, Asp571. The hydrogen bond weakened and disappeared, which may be useful to inhibitor binding.

### Principal component and free energy landscape analysis

**Fig 8:**
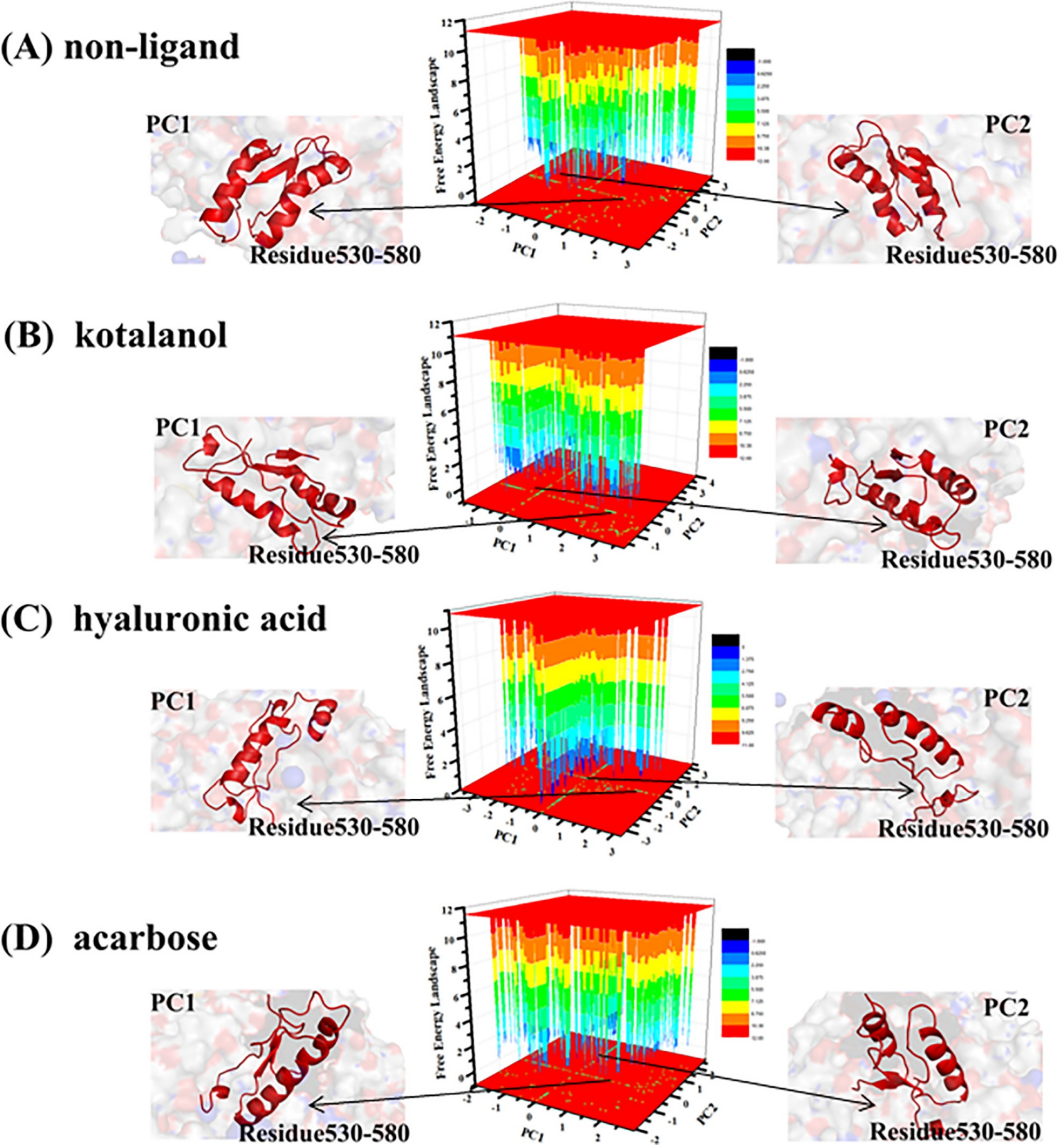
Free energy landscape (FEL) and structures of the two most stable structures of the four systems. The α helix in residue 530–580 (A) ADA. (B) ADA-FR0 complex. (C) ADA-FR2 complex. (D) ADA-PRH complex.

We probed the internal dynamics of different system, and the results were depicted in the figure. The four systems exhibited obvious differences in the correlated extents of protein motion. The Gibbs free energy landscape (FEL) was calculated using the first two principal components as reaction coordinates. Using principal component analysis (PCA), Helmoltz free energy change was calculated. The sum percentage of PC1 and PC2 for four systems were shown in Table 3, and the FELs obtained from the simulations were plotted, as shown in Fig 8A–8D. The FEL can provide remarkable information about the different conformational states accessible to the protein in the simulation. As shown in the figure, the inhibitor-protein was significantly different from the free protein. This energy minimum corresponded to a structure with some loss of irregular secondary structures, such as coils and turns. The structures of the two most stable conformations of SI revealed that the conformational changes in the α helix in residues 530–580 domain (partly disappeared) existed all SI-inhibitor complexes. This finding was consistent with the previous analysis.

**Table 3:** The probability(%) for corresponding most stable structures and substable structures.

	PC1	PC2
**non-ligand**	33.04	10.10
**kotalanol**	35.84	11.91
**HA**	25.81	11.72
**acarbose**	30.35	9.17

### Interaction of ligand and protein during the stable time and the structural motion

**Fig 9:**
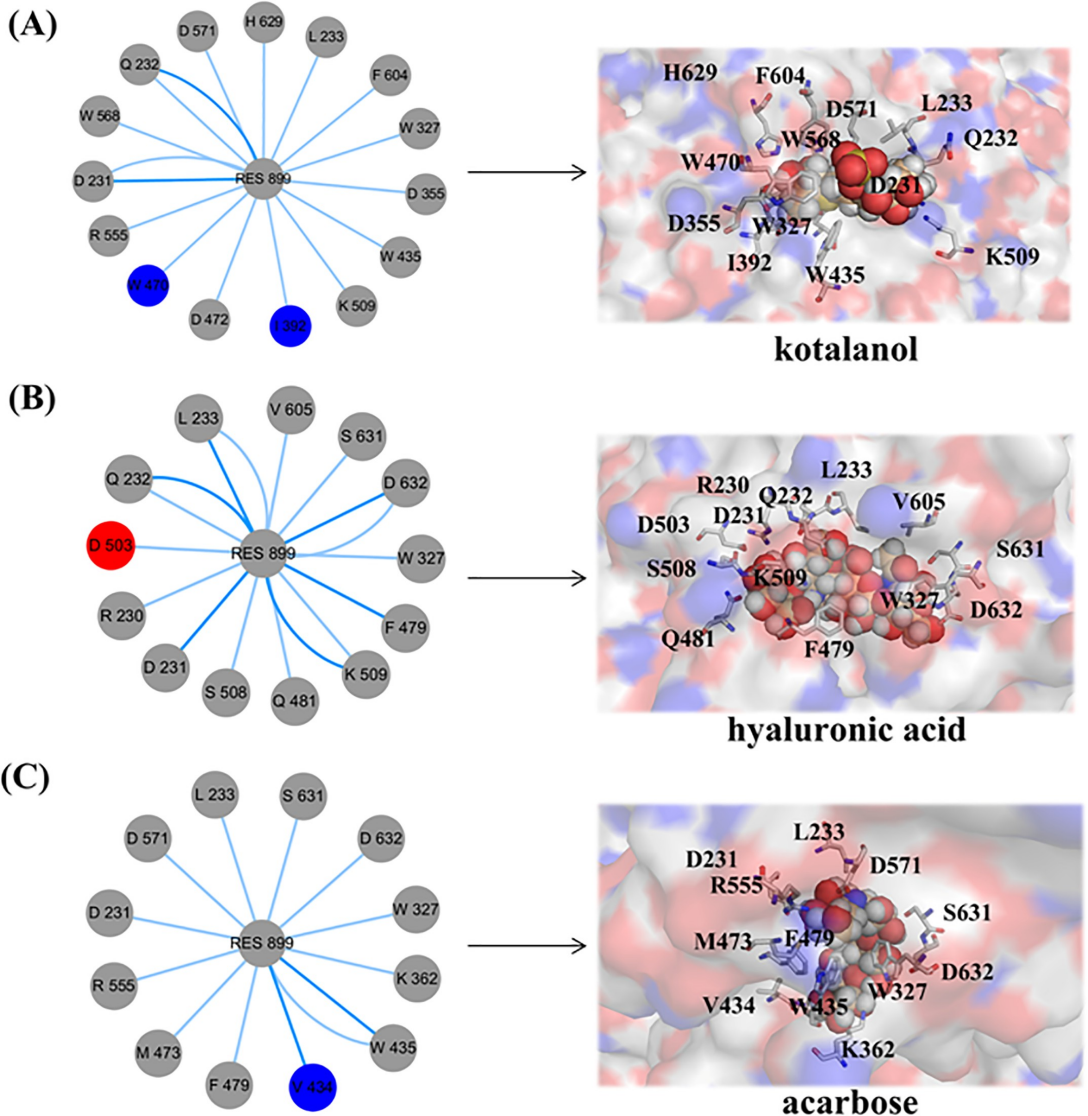
Subnetwork analysis of the protein−ligand interaction. (A) The subnetwork of SI-kotalanol. (B) The subnetwork of SI-HA. (C) The subnetwork of SI-acarbose.

Several frames were picked to obtain prospective ligand-protein interactions to compare with the binding affinities of different ligands. Nodes were coloured according to the secondary structure of the residue. For SI-kotalanol in Fig 9A, Trp470, Arg555, Asp231, Trp568, Asn232, Asp571, His629, Leu233, Phe604, Trp327, Asp355, trp455, Lys509, Ile292 and Asp472 had an interaction with kotalanol. For SI-HA in Fig 9B, Asp503, Asn232, Leu233, Val605, Ser631, Asp632, Trp327, Phe470, Lys509, Asn481, Ser508, Asp231 and Arg230 had an interaction with HA. For SI-acarbose in Fig 9C, Val434, Phe479, Met473, Arg555, Asp231, Asp571, Leu233, Ser631, Asp632, Trp327, Lys327, Lys362 and Trp435 had an interaction with acarbose. Kotalanol was the most tightly bound protein. Protein binding with acarbose and HA was weak. This finding was consistent with the previous analysis, in which HA had a higher Ki than kotalanol.

### MM-PBSA calculations

By calculating the potential energy in the vacuum, van der Waals, electrostatic interactions and net non-bonded potential energy between the protein and ligands were calculated, as shown in Table 4. An average binding energy equal to -66.47 kcal /mol was achieved for SI-kotalanol, -54.39 kcal /mol for SI-HA and -49.58 kcal /mol for SI-acarbose. This finding was consistent with the previous analysis, in which HA had a higher Ki than kotalanol.

**Table 4:** The MM-PBSA score for three complexes (kcal /mol).

Energy components	acarbose	kotalanol	HA
**ΔE**_**vdw**_	-37.49	-29.73	-29.96
**ΔE**_**ele**_	-70.21	-160.45	-64.50
**ΔE**_**MM**_	-107.66	-190.18	-94.47
**ΔG**_**polar,sol**_	56.83	52.40	44.39
**ΔG**_**nonpolar,sol**_	-29.67	-30.79	-24.24
**ΔG**_**sol**_	111.09	177.11	90.27
**ΔG**_**PB**_	83.94	155.50	70.12
**ΔG**_**binding**_	-49.58	-66.47	-54.39

## Conclusion

The HA is an important component of the *A*. *cornea (E*.*)* polysaccharide and has extremely high medicinal value. We used HA as the lead compound to search the target protein sucrase-isomaltase (SI). Then, the QSAR model was used to predict the inhibition kinetics of HA. The MD simulations showed that the binding of the three inhibitors may induce the partial disappearance of the α helix in residues 530–580. Our results may provide reasonable theoretical clues on the acidic heteropolysaccharides of *A*. *cornea (E*.*)*.

## Supporting information

S1 Fig (A) The interaction between SI and kotalanol. (B) The location diagram of crystal kotalanol and (B) docked kotalanol, (C) docked acarbose. (D) docked hyaluronic acid.
(TIF)

## References

[1] Liu T, Liu T. Preparation and Characterization of a Novel Polysaccharide-Iron(III) Complex in *Auricularia* auricula Potentially Used as an Iron Supplement. 2019;2019:6416941. doi: 10.1155/2019/6416941 . 31309110 PMC6594347

[2] Hou R, Liu X, Yan J, Xiang K, Wu X, Lin W, et al. Characterization of natural melanin from *Auricularia* auricula and its hepatoprotective effect on acute alcohol liver injury in mice. Food & function. 2019;10(2):1017–27. Epub 2019/02/02. doi: 10.1039/c8fo01624k . 30706914

[3] Zhang JC, Kong XH, Zhang PQ, Liu JN, Ma YP, Dai XD, et al. Identification of a New Fungal Pathogen Causing White Villous Disease on the Fruiting Body of the Culinary-Medicinal Mushroom *Auricularia* auricula-judae (Agaricomycetes) in China. International journal of medicinal mushrooms. 2017;19(2):155–61. Epub 2017/04/25. doi: 10.1615/IntJMedMushrooms.v19.i2.70 . 28436324

[4] Xu S, Zhang Y, Jiang K. Antioxidant activity in vitro and in vivo of the polysaccharides from different varieties of *Auricularia* auricula. Food & function. 2016;7(9):3868–79. Epub 2016/08/11. doi: 10.1039/c6fo00686h . 27506886

[5] Dong L, Liang J, Li Y, Hunang S, Wei Y, Bai X, et al. Effect of coexisting ions on Cr(VI) adsorption onto surfactant modified Auricularia auricula spent substrate in aqueous solution. Ecotoxicology and environmental safety. 2018;166:390–400. Epub 2018/10/05. doi: 10.1016/j.ecoenv.2018.09.097 . 30286398

[6] Lu LX, Yao FJ, Wang P, Fang M, Zhang YM, Zhang WT, et al. Construction of a genetic linkage map and QTL mapping of agronomic traits in Auricularia auricula-judae. Journal of microbiology (Seoul, Korea). 2017;55(10):792–9. Epub 2017/09/29. doi: 10.1007/s12275-017-7241-6 . 28956350

[7] Wang D, Jiang X, Teng S, Zhang Y, Liu Y, Li X, et al. The Antidiabetic and Antinephritic Activities of Auricularia cornea (An Albino Mutant Strain) via Modulation of Oxidative Stress in the db/db Mice. Frontiers in immunology. 2019;10:1039. Epub 2019/05/28. doi: 10.3389/fimmu.2019.01039 ; PubMed Central PMCID: PMC6517500. 31134090 PMC6517500

[8] Dai YC, Yang ZL, University BF. Notes on the nomenclature of five important edible fungi in China. Mycosystema. 2018.

[9] Saha MR, Dey P, Sarkar I, De Sarker D, Haldar B, Chaudhuri TK, et al. Acacia nilotica leaf improves insulin resistance and hyperglycemia associated acute hepatic injury and nephrotoxicity by improving systemic antioxidant status in diabetic mice. J Ethnopharmacol. 2018;210:275–86. Epub 2017/09/02. doi: 10.1016/j.jep.2017.08.036 . 28859934

[10] Salwowska NM, Bebenek KA, Żądło DA, Wcisło-Dziadecka DL. Physiochemical properties and application of hyaluronic acid: a systematic review. Journal of cosmetic dermatology. 2016;15(4):520–6. Epub 2016/06/22. doi: 10.1111/jocd.12237 . 27324942

[11] Neuman MG, Nanau RM, Oruña-Sanchez L, Coto G. Hyaluronic acid and wound healing. Journal of pharmacy & pharmaceutical sciences: a publication of the Canadian Society for Pharmaceutical Sciences, Societe canadienne des sciences pharmaceutiques. 2015;18(1):53–60. Epub 2015/04/17. doi: 10.18433/j3k89d . 25877441

[12] Papalia R, Russo F, Torre G, Albo E, Grimaldi V, Papalia G, et al. Hybrid hyaluronic acid versus high molecular weight hyaluronic acid for the treatment of osteoarthritis in obese patients. Journal of biological regulators and homeostatic agents. 2017;31(4 Suppl 2):103–9. Epub 2017/12/06. . 29202568

[13] Sim L, Willemsma C, Mohan S, Naim HY, Pinto BM, Rose DR. Structural basis for substrate selectivity in human maltase-glucoamylase and sucrase-isomaltase N-terminal domains. J Biol Chem. 2010;285(23):17763–70. Epub 2010/04/02. doi: 10.1074/jbc.M109.078980 ; PubMed Central PMCID: PMC2878540. 20356844 PMC2878540

[14] Reema Abu, Khalaf Ahmed, Mutanabbi Abdula, et al. Tryptophan and thiosemicarbazide derivatives: design, synthesis, and biological evaluation as potential β-d -galactosidase and β-d -glucosidase inhibitors. Med Chem Res. 2015.

[15] Daina A, Michielin O, Zoete V. SwissTargetPrediction: updated data and new features for efficient prediction of protein targets of small molecules. Nucleic Acids Res. 2019;47(W1):W357–w64. Epub 2019/05/21. doi: 10.1093/nar/gkz382 ; PubMed Central PMCID: PMC6602486. 31106366 PMC6602486

[16] Nguyen NT, Nguyen TH. Autodock Vina Adopts More Accurate Binding Poses but Autodock4 Forms Better Binding Affinity. 2020;60(1):204–11. doi: 10.1021/acs.jcim.9b00778 . 31887035

[17] Morris GM, Huey R, Lindstrom W, Sanner MF, Belew RK, Goodsell DS, et al. AutoDock4 and AutoDockTools4: Automated docking with selective receptor flexibility. J Comput Chem. 2009;30(16):2785–91. Epub 2009/04/29. doi: 10.1002/jcc.21256 ; PubMed Central PMCID: PMC2760638. 19399780 PMC2760638

[18] Tambi R, Morimoto G, Kosuda S, Taiji M, Kuroda Y. Large-scale all-atom molecular dynamics alanine-scanning of IAPP octapeptides provides insights into the molecular determinants of amyloidogenicity. Sci Rep. 2019;9(1):2530. Epub 2019/02/23. doi: 10.1038/s41598-018-38401-w ; PubMed Central PMCID: PMC6384915. 30792475 PMC6384915

[19] Li C, Wang J, Li Y, Chen B, Tao J, Wang X, et al. Molecular mechanisms of metal ions in regulating the catalytic efficiency of D-psicose 3-epimerase revealed by multiple short molecular dynamic simulations and free energy predictions. J Biomol Struct Dyn. 2020:1–11. Epub 2020/03/21. doi: 10.1080/07391102.2020.1737232 . 32193997

[20] Li Z, Li S, Wei X, Zhao Q. Scaled Alternating Steepest Descent Algorithm Applied for Protein Structure Determination from Nuclear Magnetic Resonance Data. J Comput Biol. 2019;26(9):1020–9. Epub 2019/04/23. doi: 10.1089/cmb.2019.0013 . 31009239

[21] Yuan G, Hu W. A conjugate gradient algorithm for large-scale unconstrained optimization problems and nonlinear equations. 2018;2018(1):113. doi: 10.1186/s13660-018-1703-1 . 29780210 PMC5945721

[22] Li Z, Xiong S, Sievers C, Hu Y, Fan Z. Influence of thermostatting on nonequilibrium molecular dynamics simulations of heat conduction in solids. 2019;151(23):234105. doi: 10.1063/1.5132543 . 31864248

[23] Heo S, Sinnott SB. Investigation of the influence of thermostat configurations on the mechanical properties of carbon nanotubes in molecular dynamics simulations. Journal of nanoscience and nanotechnology. 2007;7(4–5):1518–24. Epub 2007/04/25. doi: 10.1166/jnn.2007.335 . 17450920

[24] Quigley D, Probert MI. Langevin dynamics in constant pressure extended systems. J Chem Phys. 2004;120(24):11432–41. Epub 2004/07/23. doi: 10.1063/1.1755657 . 15268177

[25] Martonák R, Laio A, Parrinello M. Predicting crystal structures: the Parrinello-Rahman method revisited. Phys Rev Lett. 2003;90(7):075503. Epub 2003/03/14. doi: 10.1103/PhysRevLett.90.075503 . 12633242

[26] Hess B. P-LINCS: A Parallel Linear Constraint Solver for Molecular Simulation. J Chem Theory Comput. 2008;4(1):116–22. Epub 2008/01/01. doi: 10.1021/ct700200b . 26619985

[27] Boateng HA. Periodic Coulomb Tree Method: An Alternative to Parallel Particle Mesh Ewald. J Chem Theory Comput. 2020;16(1):7–17. Epub 2019/11/21. doi: 10.1021/acs.jctc.9b00648 . 31747267

[28] Zhu J, Li C, Yang H, Guo X, Huang T, Han W. Computational Study on the Effect of Inactivating/Activating Mutations on the Inhibition of MEK1 by Trametinib. Int J Mol Sci. 2020;21(6). Epub 2020/04/05. doi: 10.3390/ijms21062167 ; PubMed Central PMCID: PMC7139317. 32245216 PMC7139317

[29] Grant BJ, Rodrigues AP, ElSawy KM, McCammon JA, Caves LS. Bio3d: an R package for the comparative analysis of protein structures. Bioinformatics. 2006;22(21):2695–6. Epub 2006/08/31. doi: 10.1093/bioinformatics/btl461 . 16940322

[30] Heenan PR, Yu H, Siewny MGW, Perkins TT. Improved free-energy landscape reconstruction of bacteriorhodopsin highlights local variations in unfolding energy. 2018;148(12):123313. doi: 10.1063/1.5009108 . 29604885 PMC6910583

