# Supplementary figures and images for: Prediction of hyaluronic acid target on sucrase-isomaltase (SI) with reverse docking and molecular dynamics simulations for inhibitors binding to SI
Source: PLoS One. 2021 Jul 30;16(7):e0255351. doi: 10.1371/journal.pone.0255351 (PMC8323934; doi:10.1371/journal.pone.0255351)

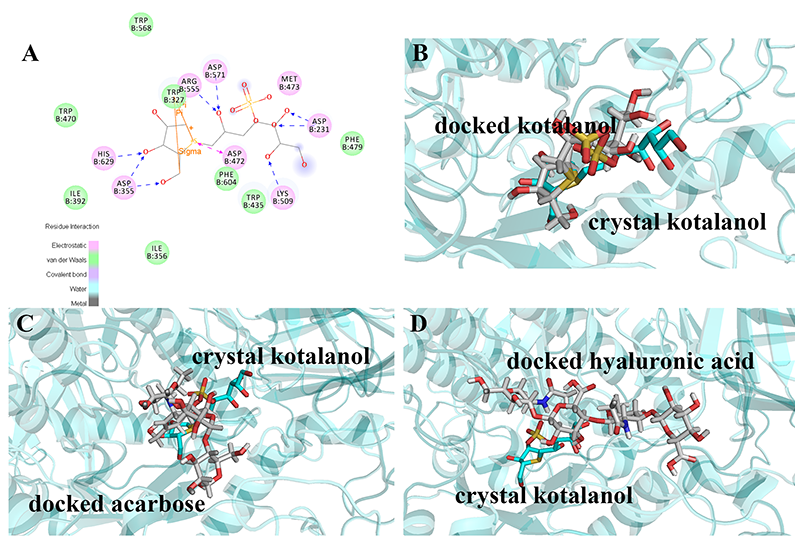

Supplement: S1 Fig — (A) The interaction between SI and kotalanol. (B) The location diagram of crystal kotalanol and (B) docked kotalanol, (C) docked acarbose. (D) docked hyaluronic acid. (TIF) [file pone.0255351.s001.tif]
